# Changes in the Properties of Different Zones in Multilayered Translucent Zirconia Used in Monolithic Restorations During Aging Process

**DOI:** 10.3390/jfb16030096

**Published:** 2025-03-10

**Authors:** Phil-Joon Koo, Jong-Hyuk Lee, Seung-Ryong Ha, Deog-Gyu Seo, Jin-Soo Ahn, Yu-Sung Choi

**Affiliations:** 1 Department of Prosthodontics, College of Dentistry, Dankook University, Cheonan 31116, Republic of Korea; philjoon0310@naver.com (P.-J.K.); hyuk928@dankook.ac.kr (J.-H.L.); hsr@dankook.ac.kr (S.-R.H.); 2 Department of Conservative Dentistry, School of Dentistry and Dental Research Institute, Seoul National University, Seoul 03080, Republic of Korea; dgseo@snu.ac.kr; 3 Department of Dental Biomaterials Science and Dental Research Institute, School of Dentistry, Seoul National University, Seoul 03080, Republic of Korea; 4 Mechanobiology Dental Medicine Research Center, Dankook University, Cheonan 31116, Republic of Korea

**Keywords:** hydrothermal aging, mechanical property, multilayered translucent zirconia, surface property, transition zone

## Abstract

This study assessed the changes in the mechanical and surface properties of the transition zone in multilayered translucent monolithic zirconia subjected to long-term hydrothermal aging. A total of 360 disk-shaped specimens (diameter: 15.0 mm; thickness: 1.2 mm) were prepared using conventional (3Y-TZP in LT; ZL, 4Y-TZP in MT; ZM) and multilayered translucent zirconia (5Y-TZP in MT Multi; ZT, 3Y/5Y-TZP in Prime; ZP) among IPS e.max ZirCAD blocks. Specimens were divided into three groups (*n* = 30) and aged in the autoclave at 134 °C under 0.2 MPa for 0 h (control group), 5 h (first aged group), and 10 h (second aged group). The mechanical and surface properties of the transition zone in the multilayered translucent zirconia were investigated, followed by statistical analysis (α = 0.05). Before and after aging, ZL (1102.64 ± 41.37 MPa) and ZP (1014.71 ± 139.86 MPa) showed the highest biaxial flexural strength (BFS); ZL showed the highest Weibull modulus (31.46) and characteristic strength (1121.63 MPa); and ZT exhibited the highest nanoindentation hardness (20.40 ± 1.80 GPa) and Young’s modulus (284.90 ± 20.07 GPa). After aging, ZL (116.75 ± 9.80 nm) exhibited the highest surface roughness (*Ra*); the monoclinic phase contents in ZL and ZP increased; and surface uplifts, microcracks, and irregular defects caused by phase transformation appeared on ZL and ZP surfaces. The 3Y/5Y-TZP transition zone exhibited flexural strength, Vickers hardness, phase distribution changes, and surface microstructure changes similar to those of 3Y-TZP before and after aging; however, the surface roughness was lower than that of 3Y-TZP and higher than those of 4Y-TZP and 5Y-TZP after aging. The mechanical and surface characteristics, excluding BFS and Vickers hardness, were influenced by the yttrium oxide content in each zone and the aging process.

## 1. Introduction

Yttria-stabilized tetragonal zirconia polycrystal (Y-TZP) was first introduced as a dental restorative material because of its tooth-like color and excellent mechanical properties [1,2]. However, Y-TZP was initially used to fabricate frameworks for veneering porcelain because of its high opacity. Nonetheless, porcelain chipping and fractures associated with the residual thermal stress of the production process have been reported as critical technical problems [3,4]. Dental zirconia consisting of 3 mol% yttrium oxide (3Y-TZP) has been developed for monolithic restorations to overcome these complications [1,2,3,4]. However, because of its opacity compared to natural teeth, it has primarily been used in the posterior regions, with limitations in the anterior teeth, where esthetics is a major consideration [5,6,7,8]. To improve transparency, strategies such as increasing the sintering temperature and reducing the amount of the sintering aid, Al_2_O_3_ can be used to reduce light scattering by grain boundaries and impurities [9]. Translucent 3Y-TZP zirconia systems were introduced in the market for dental applications, with products such as IPS e.max ZirCAD LT (Ivoclar Vivadent Inc., Schaan, Lichtenstein) and Katana HT (Kuraray Noritake Dental Inc., Tokyo, Japan); they still lack esthetics compared to glass–ceramics, and thus are somewhat limited in the anterior region.

The transparency of yttria-stabilized zirconia can also be adjusted by the cubic phase content, which can be controlled by the yttrium oxide content [9]. Increasing the yttrium oxide content leads to a larger grain size and higher cubic phase content, which are known to result in higher translucency. This is because the cubic phase is optically isotropic, successfully reducing birefringent light scattering at the grain boundaries [10,11]. Moreover, the larger grain size effectively reduces the number of grain boundaries in the material, thereby decreasing light scattering at the grain boundaries. However, higher cubic phase content induces a decrease in the proportion of the tetragonal phase, which contributes to the strength. Consequently, mechanical properties such as flexural strength and fracture toughness are compromised compared to 3Y-TZP [12,13,14]. In other words, the mechanical performance and translucency of dental zirconia exhibit a trade-off relationship, and thus have a wide range of clinical applications [15,16,17]. Translucent monolithic zirconia ceramics with increased yttrium oxide content have been successfully developed to improve transparency and esthetics. Different grades are available for monolithic restorations, namely 4 mol% yttria partially stabilized zirconia (Y-PSZ) and 5Y-PSZ. Higher yttrium oxide content, such as 4 or 5 mol%, enhances the translucency of zirconia ceramics and is called third-generation dental zirconia.

Recently, multilayered translucent zirconias have been introduced to further improve the optical properties of dental restorative materials, which may be more suitable for anterior region applications [18]; IPS e.max ZirCAD MT Multi (Ivoclar Vivadent Inc., Schaan, Lichtenstein), Katana STML, and Katana UTML (Kuraray Noritake Dental Inc, Tokyo, Japan) are examples of these systems. It has been reported that multilayered translucent zirconia ceramics exhibit enhanced color reproduction capabilities in comparison to traditional translucent zirconia ceramics [19,20,21]. The aim was to reproduce the shade gradient observed in natural teeth, considering the presence of both enamel and dentin. Multilayered translucent zirconia ceramics consist of three layers: incisal, transitional, and dentin. The incisal region of the restoration is the most translucent, and the opacity and chroma increase toward the cervical region.

Several studies have reported that the optical properties of multilayered translucent zirconia ceramics are inferior to those of lithium disilicate ceramic [22,23,24]. Nevertheless, the utilization of multilayered translucent zirconia ceramics has increased in dental prosthetic restorations because of their remarkable translucency and esthetic properties [25,26,27]. However, the various properties of multilayered translucent zirconia should be stable for reliable and successful use in oral cavities. The mechanical properties and surface characteristics of dental zirconia are affected by hydrothermal aging [28,29,30,31].

The stability of dental zirconia is crucial during aging and low-temperature degradation (LTD) under long-term hydrothermal stress [32]. LTD is a tetragonal to monoclinic phase (*t*⟶*m*) transformation of zirconia and occurs when there is prolonged hydrothermal stress from synovial fluid, water, and blood [15]. This causes microcrack formation and surface uplift/roughening to release stress due to the phase transformation accompanied by volume expansion. LTD also degrades hardness, fracture toughness, and flexural strength, which may lead to deterioration [33,34,35]. Hydrothermal treatment in an autoclave is an accelerated method for aging that simulates long-term application in an oral environment. Previous studies indicate that 1 h of autoclaving is approximately equivalent to 3–4 years of in vivo aging [32,36]. To ensure successful clinical application of multilayered translucent zirconia ceramics, it is important to analyze the effects of hydrothermal aging in a long-term environment similar to that of the oral cavity.

Several studies have reported variations in the mechanical properties and surface characteristics of translucent monolithic zirconia after aging processes [37,38,39,40,41]. However, these studies focused on properties of materials, limited types, and short-term aging. In conventional multilayered translucent zirconia ceramics, the 3Y-TZP and 5Y-TZP regions are stacked and combined. The multilayered translucent zirconia ceramic system used in this study was IPS e.max ZirCAD Prime. Unlike the general case in which the yttrium oxide content is uniform in the transition zone, IPS e.max ZirCAD Prime utilizes the latest technology from Ivoclar Vivadent, known as gradient technology. This involves creating a transition zone by blending different raw materials, specifically 3Y-TZP and 5Y-TZP. However, the changes in various long-term properties of different zones in multilayered translucent zirconias used in monolithic restorations during aging have been rarely explored.

Therefore, this study was undertaken to assess the alterations in the mechanical properties and surface characteristics of the transition zone (3Y/5Y-TZP) in multilayered translucent monolithic zirconia, fabricated using gradient technology, under prolonged hydrothermal aging conditions. Furthermore, the long-term stability of the various properties of this transition zone of multilayered translucent zirconia was compared with those of different zones in conventional and multilayered translucent monolithic zirconia ceramics consisting of 3Y-, 4Y-, and 5Y-TZP. The three null hypotheses in the present study were as follows: (i) the properties of different zones in translucent zirconia ceramics would not be affected by aging; (ii) the yttrium oxide content would not affect the properties of different zones in translucent zirconia ceramics; and (iii) the properties of different zones in translucent zirconia ceramics after hydrothermal aging would not be affected by the yttrium oxide content.

## 2. Materials and Methods

### 2.1. Specimen Preparation

Four IPS e.max ZirCAD (Ivoclar Vivadent Inc., Schaan, Lichtenstein) systems were investigated. Two conventional translucent zirconia ceramics (3Y-TZP in IPS e.max ZirCAD LT; ZL, 4Y-TZP in IPS e.max ZirCAD MT; ZM) and two multilayered translucent zirconia ceramics (5Y-TZP in IPS e.max ZirCAD MT Multi; ZT, 3Y/5Y-TZP in IPS e.max ZirCAD Prime; ZP) were used (Table 1) for evaluating the mechanical and surface properties of the transition zone in multilayered translucent monolithic zirconia. ZT and ZP are multilayered zirconia blocks engineered to mimic the natural gradient and translucency of dental enamel and dentin by incorporating distinct incisal, transition, and dentin zones. The ZT variant employs a composition wherein the incisal region consists of highly translucent 5Y-TZP to enhance optical properties, while the dentin region is composed of more opaque 4Y-TZP, effectively replicating the opacity characteristic of natural dentin. Conversely, ZP is manufactured through a blending process of 3Y-TZP and 5Y-TZP, resulting in a unique combination of mechanical strength and esthetic properties tailored for dental restorations. In this study, ZT specimens were obtained from the 5Y-TZP incisal zone, while ZP specimens were derived from the 3Y/5Y-TZP transition zone. The zirconia specimens were classified according to their respective yttria content to facilitate a comprehensive analysis of their structural and functional characteristics.

For each group, 90 specimens were prepared to evaluate the mechanical and surface properties, resulting in a total of 360 specimens. Each zirconia specimen was designed to be disk shaped, using a computer-aided design (CAD) program (Solidworks, Dassault Systèmes, Paris, France) and fabricated using a precision milling machine (Cameleon CS, Neobiotech Co., Seoul, Korea) with a thickness and diameter of 3.0 and 15.0 mm, respectively. ZT specimens were placed in the incisal zone. Conversely, ZP specimens were designed to be located in the transition zone. Each zirconia specimen was sintered at 1500 °C according to the manufacturer’s instructions, and a 20% shrinkage rate was considered.

All the specimens were then processed to form shapes, each with a diameter and thickness of 15.0 and 1.2 mm, respectively, and were polished on both sides by grinding (SPL-15 Grind X, Okamoto Co., Osaka, Japan) with the 6 μm diamond slurry (Hyprez, Engis, Wheeling, IL, USA) and 1 μm grit size to ensure the uniform dimension according to ISO 6872:2015 [42].

After grinding and polishing, the size of each specimen was measured with a digital caliper (Absolute 500; Mitutoyo Co., Kawasaki, Japan). Following that, heat treatments using a furnace (Programat EP 5000, Ivoclar Vivadent, Schaan, Lichtenstein) were conducted for 1 h in a temperature range of 900–1000 °C, in order to induce the reverse phase transformation from a monoclinic to tetragonal phase. Each zirconia specimen was ultrasonically cleaned with distilled water and dried at room temperature (21–23 °C) for 24 h.

### 2.2. Hydrothermal Aging Process

Thirty specimens were randomly selected from each group for evaluation of their mechanical properties and surface characteristics. One subgroup was immersed in distilled water at room temperature for 24 h, dried, and used as a control. The other two experimental subgroups went through the hydrothermal aging process using an autoclave (Hiclave H480S, Hongik Medical Systems, Ansan, Republic of Korea) with distilled water at 134 °C under 0.2 MPa for 5 and 10 h (Table 2).

### 2.3. Measurements and Analyses of Mechanical Properties

Fifteen specimens from each group were subjected to the BFS test in accordance with ISO 6872:2015 [41]. The universal testing machine (Instron 5583, Instron Co., Norwood, MA, USA) and a 5 kN load cell were applied. The specimens were held by placing three hardened steel balls with diameters of 3 mm on the sample holder, arranged at a 120° angle, which provided a support circle with a diameter of 11 mm. Each disc-shaped specimen was placed in the center of three balls and loaded with a flat pin (1.5 mm diameter and 2 mm length) at a crosshead speed of 0.5 mm/min. The load was applied to measure the maximum fracture load of each specimen. The fracture load was measured in N, and the flexural strength (*σ*) was calculated in MPa with the following equation:

(1) $$\sigma \;=\frac{-0.2387P\left(X-Y\right)}{b^{2}}$$

(2) $$X=\left(1+v\right)\ln{\left(\frac{r_{2}}{r_{3}}\right)}^{2}+\left[\frac{\left(1-v\right)}{2}\right]{\left(\frac{r_{2}}{r_{3}}\right)}^{2}$$

(3) $$Y=\left(1+v\right)\left[1+\ln{\left(\frac{r_{1}}{r_{3}}\right)}^{2}\right]+\left(1-v\right){\left(\frac{r_{1}}{r_{3}}\right)}^{2}$$

where *b* is the specimen height (mm), *v* is Poisson’s ratio (*v* = 0.3), *r*_1_ is the radius of the support circle (mm), *r*_2_ is the radius of the flat pin (mm), and *r*_3_ is the radius of the specimen (mm).

The Weibull modulus (*w*) and the characteristic strength (*σ*_0_) were calculated to assess the reliability of the specimens, based on the BFS (*σ*) in accordance with ISO 6872:2015 [41]. First, the *σ* values were arranged in ascending order and calculated using the following equation:

(4) $$P_{f}=\;\frac{i-0.5}{N}$$

where *P_f_* is the fracture probability between 0 and 1, *N* is the number of specimens, and *i* is the ascending order of strength. The sorted data were drawn on the graph using ln σ and ln [1/(1 − *P_f_*)] as the *x*-axis and *y*-axis, respectively. The w was calculated from the slope of the graph and the σ0 was determined from the y-intercept.

The nanoindentation hardness and Young’s modulus were analyzed on the polished surfaces of the specimens using a 40 nm Berkovich diamond by iNano Nanoindenter (Nanomechanics Inc., Oak Ridge, TN, USA). The maximum penetration depth was applied to 2 µm, and the required load was set to 45 mN. The Poisson’s ratio was 0.3 [43]. The results were evaluated using TestWorks software (version 3.3.1, Nanomechanics Inc.).

Vickers hardness was measured via a microhardness tester (HM-220B, Mitutoyo, Kawasaki, Japan). Three indentations were made on each specimen at a loading mass of 1 kgf (9.8 N). The time of force application was set to 10 s, and the resting time between applications on the same sample was set to at least 30 s. The indentation diagonal length (mm) was determined using AVPAK System B software (20 V3.0, Mitutoyo). Microhardness was calculated using the following equation:

(5) $$H_{v}=1.8544\;F/d^{2}$$

where *Hv* (VH) is the Vickers hardness value at the applied force *F* (kgf), and *d* is the mean length of the indentation diagonal (mm).

### 2.4. Measurements and Analyses of Surface Properties

Confocal laser scanning microscopy (CLSM; LSM 880, Carl Zeiss, Oberkochen, Germany) was used to analyze the surface roughness (*Ra* and *Rq*) of the specimens. A 543 nm helium-neon laser (1 mW) was applied as the light source, and each specimen was measured at 20× magnification. The measuring area was 300 × 300 µm^2^, and the CLSM images were observed using a Zeiss LSM Image Examiner (version 3.1, Carl Zeiss).

A representative specimen of each subgroup was subjected to phase transformation analyses by X-ray diffraction (XRD; SmartLab, Rigaku Co., Tokyo, Japan). XRD data were obtained by means of the Bragg–Brentano geometry in 2θ range of 24–36° with Kα radiation via XRD. The scan was performed at 30 mA and 40 kV, with a step size of 0.005°/step, scanning rate of 0.25°/min, and scan time of 8 s/step. The monoclinic phase peak intensity ratio (*X_m_*) was calculated using the equation proposed by Garvie and Nicholson [44]:

(6) $$X_{m}=\frac{\left[I_{m}\left(\bar{1}11\right)+I_{m}\left(111\right)\right]}{\left[I_{m}\left(\bar{1}11\right)+I_{m}\left(111\right)+I_{t}\left(111\right)\right]}$$

where *I_m_* and *I_t_* indicate the integrated intensities of the monoclinic ($\bar{1}11)$, (111), and tetragonal (111) peaks at approximately 28°, 31°, and 30°, respectively. The volume fraction of the monoclinic phase (*F_m_*) was analyzed using the method proposed by Toraya et al. [45]:

(7) $$F_{m}=\;\frac{1.311X_{m}}{1+0.311X_{m}}$$

Microstructural analyses were conducted using a field emission scanning electron microscope (FE-SEM; SU8230, Hitachi High Technologies Corp., Tokyo, Japan). The surfaces of the specimens were analyzed via the FE-SEM at 30,000× *g* magnification. The analyzed voltage was set to 5.0 kV.

Energy dispersive X-ray spectroscopy (EDS; SU8230, Hitachi High Technologies Corp., Tokyo, Japan) analyses were performed to evaluate the changes in the elemental composition (Zr, O, Y, Al, and Hf) during the aging process at the accelerating voltage of 15.0 kV and 200× *g* magnification.

### 2.5. Statistical Analyses

Statistical analyses were conducted using statistical software (SPSS Statistics for Windows v23.0, IBM Corp, Armonk, NY, USA). Data normality was analyzed using the Shapiro–Wilk test. One-way ANOVA was performed to compare the mechanical properties and surface characteristics between each material and Tukey’s honestly significant difference (HSD) test for post hoc analysis (α = 0.05). An independent samples *t*-test was used to analyze the variables related to the mechanical and surface properties during the aging process. Two-way ANOVA and Tukey’s HSD post hoc tests were performed to analyze the interaction between material types categorized by yttrium oxide content in each zone and aging (α = 0.05).

## 3. Results

### 3.1. Comparative Analyses of Mechanical Properties

**Figure 1:**
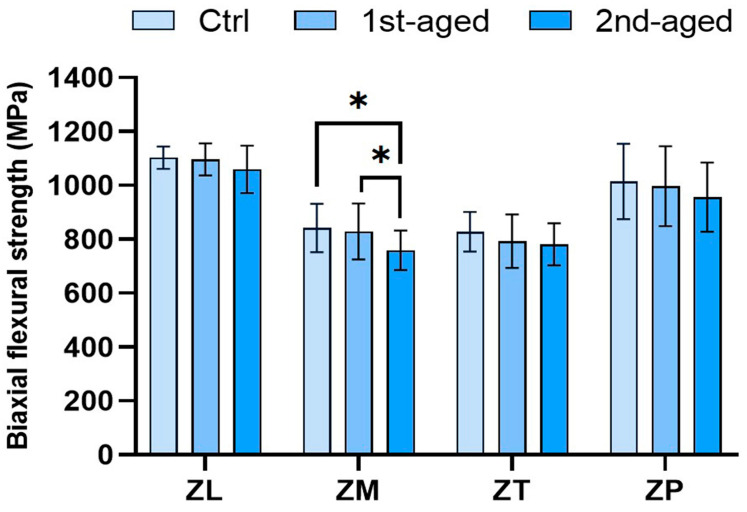
Mean ± standard deviation values and statistical analysis of biaxial flexural strength. * denotes a significant difference at *p* < 0.05.

The BFS values of all the groups are presented in Figure 1 and Table S1. Before aging, the highest values were shown by ZL (1102.64 ± 41.37 MPa) and ZP (1014.71 ± 139.86 MPa), whereas the lowest were shown by ZM (841.99 ± 89.67 MPa) and ZT (827.94 ± 73.86 MPa). In the first aged group, the highest values were shown by ZL (1096.20 ± 59.17 MPa) and ZP (996.91 ± 147.67 MPa), whereas the lowest were shown by ZM (829.39 ± 103.81 MPa) and ZT (792.48 ± 99.15 MPa). In the second aged group, ZL (1059.53 ± 88.29 MPa) and ZP (968.33 ± 128.64 MPa) showed the highest values, whereas the lowest values were shown by ZT (781.09 ± 77.77 MPa) and ZM (758.69 ± 73.53 MPa). The values shown by ZL and ZP were significantly higher than those of ZM and ZT before and after the aging process (*p* < 0.05).

**Figure 2:**
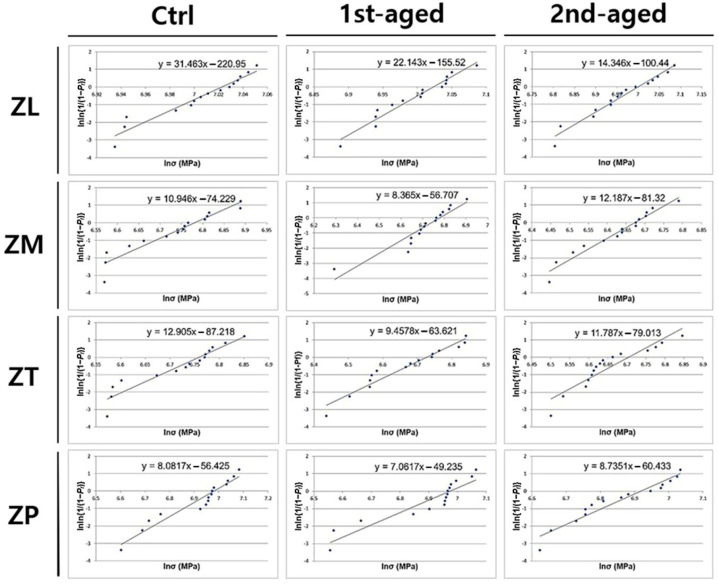
Probability plot for Weibull analysis for all the groups.

Figure 2 presents the Weibull analysis results of all the groups. The Weibull moduli and characteristic strengths of all groups are presented in Table S2. Before aging, the Weibull moduli decreased in the order of ZL (31.46, maximum value) > ZT > ZM > ZP (8.08, minimum value). After the first aging, ZL (22.14) showed the highest value, followed by ZT and ZM, and ZP (7.06) exhibited the lowest value. After the second aging, ZL (14.35) showed the highest value, whereas ZP (8.74) showed the lowest value. All groups exhibited a decreasing tendency as the aging time increased. Before aging, the characteristic strength decreased from a maximum at ZL (1121.63 MPa) through ZP and ZM to a minimum at ZT (861.32 MPa). After the first aging, ZL (1122.64 MPa) exhibited the highest value, whereas ZT (834.50 MPa) exhibited the lowest. After the second aging, ZL (1098.01 MPa) exhibited the highest value, whereas ZM (790.51 MPa) exhibited the lowest value. All groups showed a decreasing tendency as the aging time increased.

**Figure 3:**
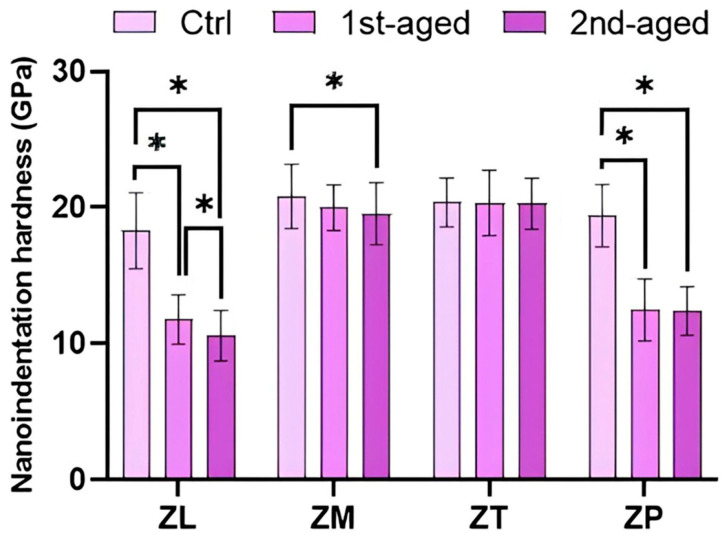
Mean ± standard deviation values and statistical analysis of nanoindentation hardness. * denotes a significant difference at *p* < 0.05.

The nanoindentation hardness values of all the groups are presented in Figure 3 and Table S3A. Before aging, the highest values were shown by ZM (20.82 ± 2.37 GPa) and ZT (20.40 ± 1.80 GPa), whereas ZL (18.30 ± 2.79 GPa) exhibited the lowest value. In the first aged group, the highest values were still shown by ZT (20.34 ± 2.41 GPa) and ZM (19.98 ± 1.67 GPa), whereas the lowest values were obtained from ZL (11.79 ± 1.80 GPa) and ZP (12.48 ± 2.28 GPa). In the second aged group, the highest values were shown by ZT (20.29 ± 1.86 GPa) and ZM (19.54 ± 2.27 GPa), whereas the lowest value was shown by ZL (10.61 ± 1.87 GPa). ZT exhibited the highest values before and after aging (*p* < 0.05).

**Figure 4:**
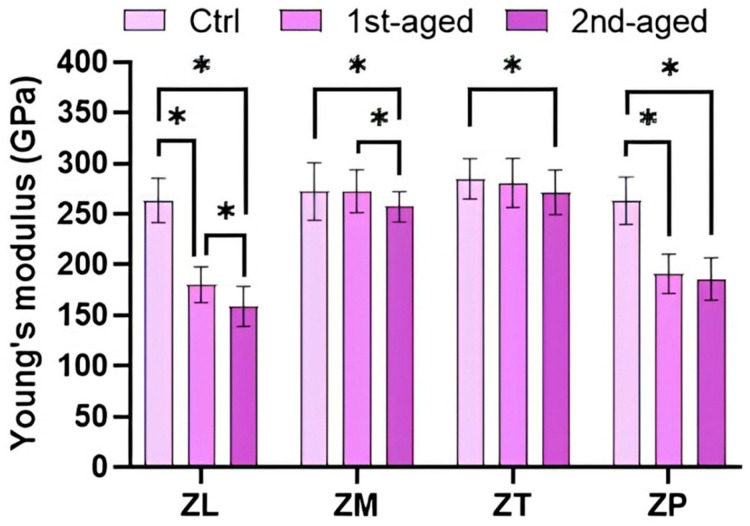
Mean ± standard deviation values and statistical analysis of Young’s modulus. * denotes a significant difference at *p* < 0.05.

Young’s moduli of all the groups are presented in Figure 4 and Table S3B. Before aging, the highest value was shown by ZT (284.90 ± 20.07 GPa), whereas the lowest values were shown by ZL (263.24 ± 21.81 GPa) and ZP (263.29 ± 23.32 GPa). After first aging, the highest values were shown by ZM (272.29 ± 21.28 GPa) and ZT (280.80 ± 24.38 GPa), whereas the lowest values were shown by ZL (180.30 ± 17.49 GPa) and ZP (191.18 ± 19.37 GPa). After the second aging, the value decreased in the order of ZT (271.23 ± 21.85 GPa), ZM (257.01 ± 14.83 GPa), ZP (185.99 ± 20.69 GPa), and ZL (158.82 ± 20.09 GPa). All the groups exhibited a decreasing tendency with increasing aging time. ZT exhibited the highest values before and after aging (*p* < 0.05).

**Figure 5:**
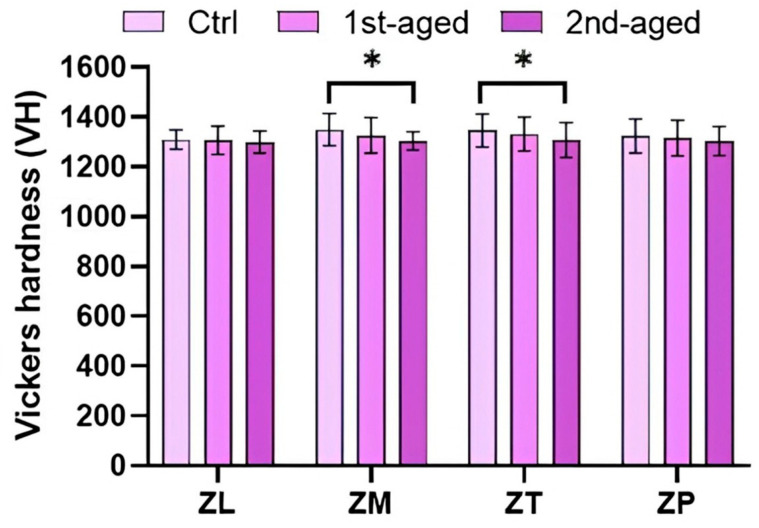
Mean ± standard deviation values and statistical analysis of Vickers hardness. * denotes a significant difference at *p* < 0.05.

Vickers hardness values of all the groups are presented in Figure 5 and Table S4. Before aging, the highest values were shown by ZM (1349.24 ± 64.14 VH) and ZT (1345.64 ± 66.18 VH), whereas the lowest value was shown by ZL (1308.86 ± 39.39 VH). After the first and second agings, there was no significant difference among ZL, ZM, ZT, and ZP (*p* > 0.05).

### 3.2. Comparative Analyses of Surface Properties

**Figure 6:**
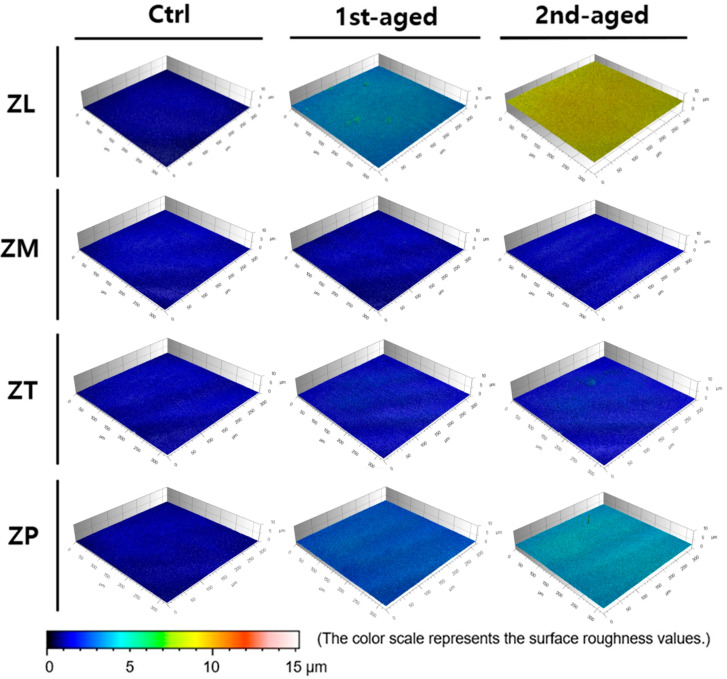
Representative CLSM images of all the groups.

Representative CLSM images of all the groups are presented in Figure 6 and Table S5.

**Figure 7:**
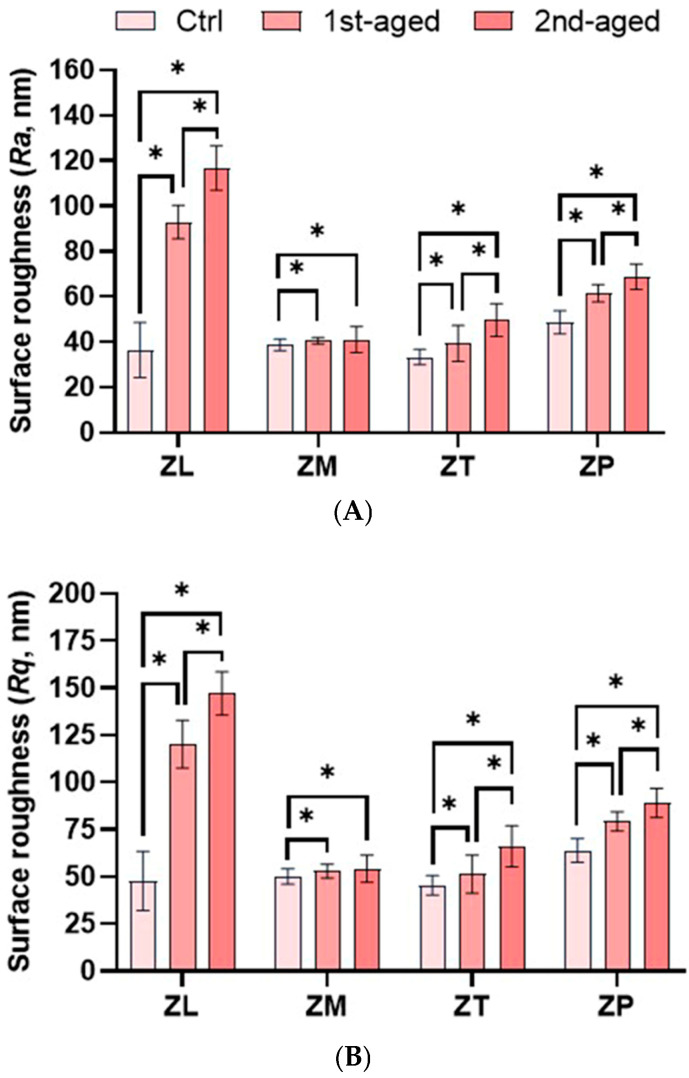
Mean ± standard deviation values and statistical analysis of surface roughness of all specimens in the groups. (**A**) *Ra*; (**B**) *Rq*. *denotes a significant difference at *p* < 0.05.

Figure 7 presents the mean values, standard deviations, and statistical analysis results of the surface roughness (*Ra* and *Rq*) using CLSM. In the different zones of all the materials, the *Ra* and *Rq* values were the lowest for all the control groups (*p* < 0.05). In the different zones of all the materials, the surface roughness values (*Ra* and *Rq*) were the lowest for all the control groups (*p* < 0.05). Before aging, the highest *Ra* value was shown by ZP (48.69 ± 5.09 nm), whereas the lowest values were detected from ZL (36.45 ± 12.10 nm) and ZT (33.29 ± 3.42 nm). After the first aging, the highest *Ra* value was shown by ZL (92.95 ± 7.41 nm), whereas the lowest values were exhibited by ZM (40.54 ± 1.39 nm) and ZT (39.34 ± 7.95 nm). After the second aging, ZL (116.75 ± 9.80 nm) showed the highest *Ra* value, whereas ZM (41.08 ± 5.84 nm) exhibited the lowest value (*p* < 0.05). The *Rq* values exhibited a similar trend to *Ra*. The *Ra* and *Rq* values increased in all groups after aging (*p* < 0.05).

**Figure 8:**
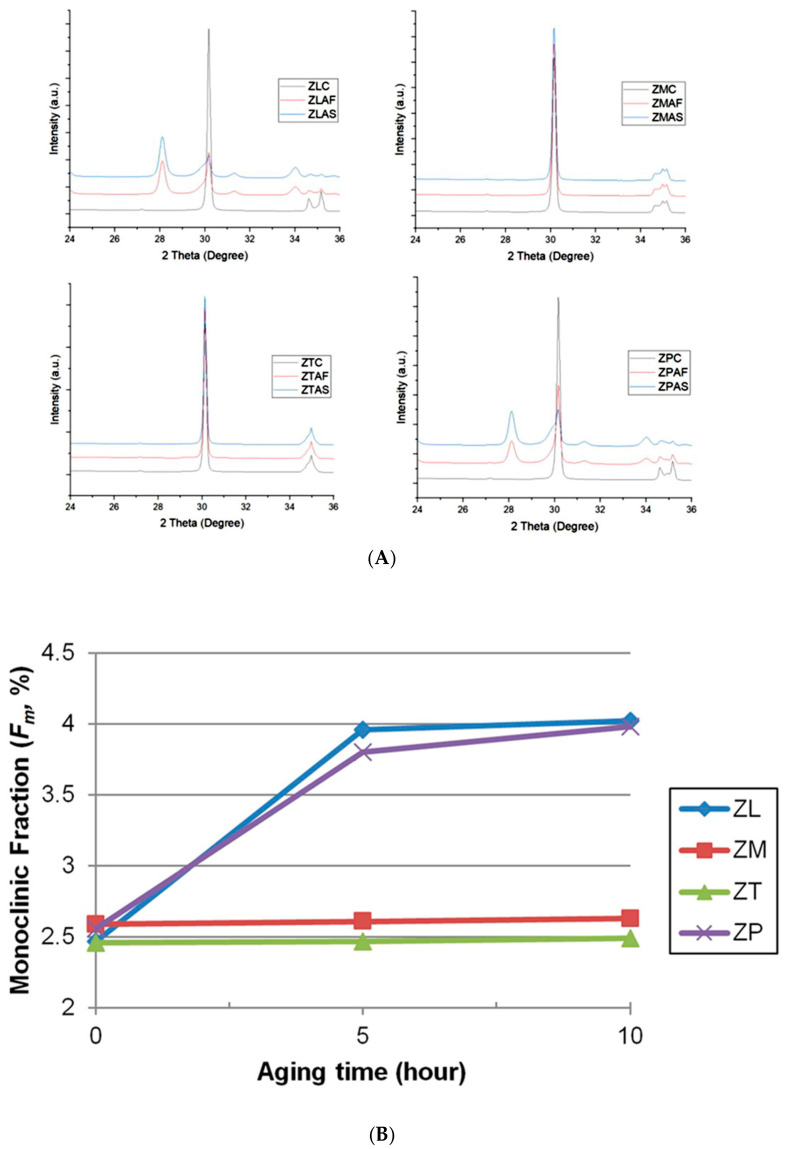
Phase transformation analyses in accordance with aging time. (**A**) Representative XRD patterns; (**B**) monoclinic volume fraction (*F_m_*).

The XRD data for the different zones of all the materials are presented in Figure 8A. The tetragonal phase (*t*-phase) was the major crystalline phase observed in the control group. The diffraction peak positions for the *t*-phase were similar in the different zones of the multilayered and conventional groups. A monoclinic phase (*m*-phase) was detected on the surface of the aged specimens with the strained *t*-phase. Hydrothermal aging primarily induced an increase in the monoclinic ($\bar{1}11)$ peak, representing the most stable monoclinic phase, and simultaneously triggered the transformation from a tetragonal to monoclinic phase (*t*⟶*m*), inducing the increase in the monoclinic (200) peak. After aging, the XRD analyses of all groups for different durations revealed a gradual increase in the monoclinic phase (*m*-phase) of the four zirconia materials compared with the *t*-phase as the aging time increased. Figure 8B and Table S6 present the analysis of *F_m_* according to aging time from the XRD data. Before aging, the *F_m_* values were ZL (2.47%), ZM (2.59%), ZT (2.46%), and ZP (2.56%). After the first aging, the highest value was observed in ZL (3.96%), followed by ZP (3.80%), ZM (2.61%), and ZT (2.47%). After the second aging, the highest value was observed for ZL (4.02%), followed by ZP (3.98%), ZM (2.63%), and ZT (2.49%). Throughout the aging process, the monoclinic phase tended to increase in all the groups. ZL and ZP exhibited an evident increase in monoclinic phase content after the first aging.

**Figure 9:**
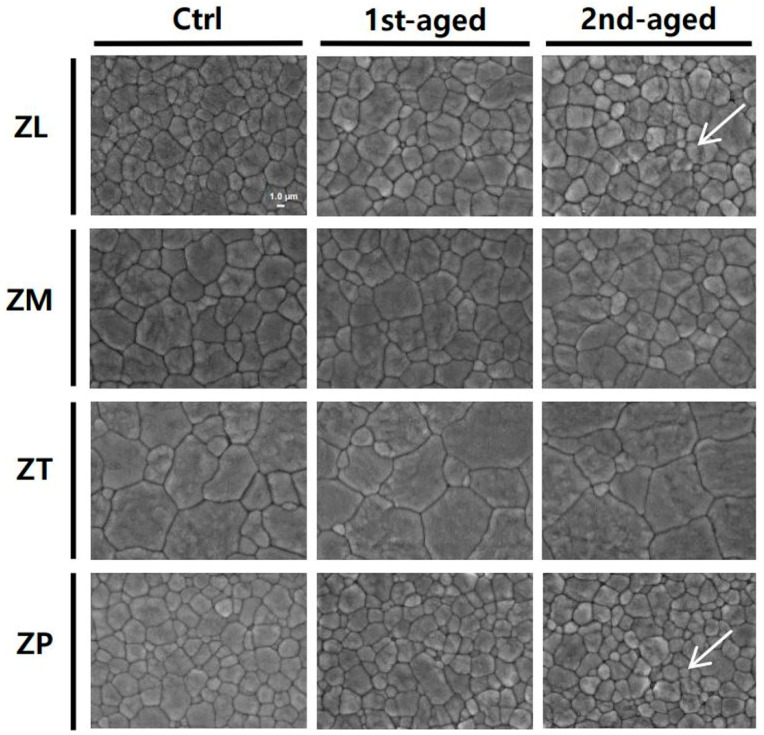
FE-SEM images showing the surface topography of all the specimens in the groups. The white arrows indicate irregular defects, microcracks, and surface uplifts after aging.

The FE-SEM images showing surface topographies of all the groups are presented in Figure 9. As aging progresses, the increase in surface roughness could be observed owing to microcracks, grain push-out, and surface uplift caused by phase transformation. All tested materials showed irregular defects, microcracks, and surface uplifts after aging, which were clearly visible on the surfaces of the ZL and ZP groups. In addition, as the yttria content increased, the grain size increased, and changes in surface particles were not as evident as in groups ZL and ZP.

**Figure 10:**
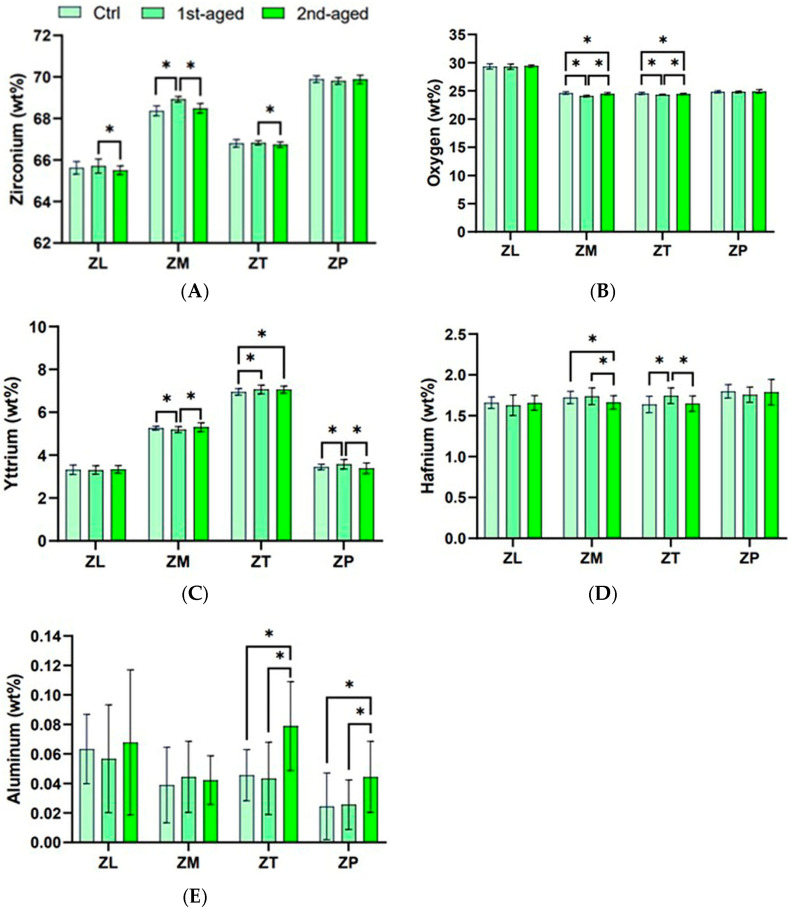
Elemental analysis. (**A**) Zirconium; (**B**) oxygen; (**C**) yttrium; (**D**) hafnium; and (**E**) aluminum. * denotes a significant difference at *p* < 0.05.

Comparing the surface compositions during the aging process in all the groups, several elements presented statistically significant differences among the materials, as presented in Figure 10 and Table S7A–D. All groups had the highest percentages of Zr (maximum value), O, Y, Hf, and Al (minimum value), in that order. The results of the independent samples *t*-test indicated variations in the contents of various elements. ZL showed no significant changes in composition after aging. However, after the second aging, Zr levels significantly decreased (*p* = 0.010). ZM showed no significant difference in Al content, whereas the other components varied widely. ZT showed significant differences among the O groups, whereas the remaining components showed varying degrees of change. ZP showed no significant changes in O, Zr, or Hf during the aging process, whereas only Al and Y showed changes.

### 3.3. Statistical Analyses of 2-Way ANOVA of All Groups

Two-way ANOVA results of all groups for mechanical and surface properties values are exhibited in Table S8A–D. Significant interactions were not detected between the aging process and yttrium oxide contents of different zones for the mechanical properties of biaxial flexural strength and Vickers hardness (*p* > 0.05). However, there were significant interactions among the aging process and yttrium oxide contents of different zones for the mechanical and surface properties of the nanoindentation hardness, Young’s modulus, the surface roughness, and elemental analysis (*p* < 0.05).

## 4. Discussion

In this study, we assessed the variations in the mechanical properties and surface characteristics of the transition zone in multilayered translucent monolithic zirconia ceramics subjected to long-term hydrothermal aging and compared the results with those of zones with different yttrium oxide contents. We determined that the mechanical properties and surface characteristics of ZP changed after aging. Therefore, the first null hypothesis was rejected, in which hydrothermal aging would not affect the properties of different zones in translucent monolithic zirconia ceramics. A previous study reported that increasing the yttrium oxide content led to higher cubic phase content [9]. A higher cubic phase content induces a decrease in the proportion of the tetragonal phase, which contributes to the strength. Consequently, mechanical properties such as flexural strength and fracture toughness are compromised compared to 3Y-TZP [12,13,14]; this result was validated by our analyses. Therefore, the second null hypothesis was also rejected, in which the yttrium oxide content would not affect the properties of different zones in translucent monolithic zirconia. Finally, the third null hypothesis was rejected, in which the yttrium oxide content would not affect the properties of different zones in translucent monolithic zirconia ceramics after hydrothermal aging. Several studies have reported variations in the mechanical properties and surface characteristics of multilayered translucent zirconia during the aging process [25,26,27]. 3Y-TZP is susceptible to hydrothermal aging, during which a spontaneous *t*⟶*m* transformation occurs in the humid environment at moderate temperatures, including in the human body. From this perspective, zirconia stabilized with the normally higher yttrium oxide content has higher aging stability, lowering the associated risks of damaging the mechanical properties and surface roughening [27,33]. In other words, as the yttrium oxide content increased, the effect of hydrothermal aging decreased. This result is consistent with those of the present study.

Aging is a slow progression. Currently, there is no recognized mechanism for aging zirconia ceramics at body temperature [38]. Evidence of the aging of zirconia ceramics has been reported in various experiments [32,33]. Thermocycling can be used to stimulate LTD, which represents intraoral temperature changes caused by routine drinking, eating, and breathing [46]. However, the lack of a standardized protocol makes it difficult to compare the results [47,48]. ISO/TS 11405 recommends using two water baths with a temperature of 5–55 °C and a dwell time of at least 20 s. However, regulatory mechanisms tend to restore the temperature to 37 °C in the oral cavity, and patients do not tolerate this extreme stimulus for long periods. Instead, LTD of zirconia is typically performed in a steam chamber or autoclave, where the elapsed time, temperature, and steam pressure are controlled variables. Autoclave treatment is the most effective method for accelerating the aging process. Steam sterilization at 134 °C for 5 h is suggested to reproduce degradation equivalent to 15 to 20 years at 37 °C [1,32]. After aging, for zirconia ceramics, less than 25% of the *t*⟶*m* transformation is expected, which is an acceptable level in accordance with ISO 13356:2015 [30]. In this study, it was observed that *t*⟶*m* transformation also increased as the aging time increased, changing the monoclinic fraction of ZL and ZP after 5 h of aging. However, it was 3.96% for ZL and 3.80% for ZP, which were well below 25%. The aging process occurs very slowly in the oral environment; however, translucent zirconia ceramics gradually undergo degradation. Declines in strength due to aging can be exacerbated by repeated thermal stress and chewing forces [35]. Assessing the additional effects of aging on zirconia is critical for the successful clinical application of multilayered translucent zirconia ceramics, as these materials are subjected to hydrothermal cyclic loading within the oral environment. In particular, it is necessary to understand how the physical properties of the 3Y/5Y-TZP transition zone produced by gradient technology change before and after aging compared with those of 3Y-, 4Y-, and 5Y-TZP.

Flexural strength is important because of the high degree of brittleness [49]. The BFS test, conducted using a universal testing machine, is similar to situations in which stress is concentrated on the surface of a tooth or during dental restoration in the oral environment [49]. In this study, the BFS values of ZL and ZP were significantly higher than those of ZM and ZT before and after aging. ZP exhibited results similar to those of ZL. All the zirconia specimens exhibited a decreasing tendency as the aging time increased. However, no significant differences were observed after hydrothermal aging, except in the case of ZM. This result was consistent with a previous result that compared the BFS of dental zirconia showing different degrees of translucency after autoclave treatment at 134 °C and 0.2 MPa for 2, 6, 18, and 54 h [19]. Therefore, based on these findings, it can be inferred that the flexural strength of translucent monolithic zirconia is unlikely to be significantly influenced by prolonged exposure to oral conditions, suggesting its potential stability over long periods of clinical use.

The brittle zirconia did not exhibit a normal strength value distribution with respect to the mean. This can be explained as the distribution of the probability of failure occurring at a given stress. The Weibull modulus was used as a standard to indicate the reliability of the material [50]. ZL had the highest Weibull modulus, whereas ZP had the lowest. This was likely due to the inhomogeneous grain size distribution of ZP and wider distribution of grain sizes, which increased the probability of failure [15]. For the characteristic strength, at the failure probability of approximately 63%, ZL still showed the highest value before and after aging, whereas ZT and ZM showed the lowest values after the first and second aging processes, respectively. This showed a tendency similar to that of the BFS. The result is consistent with a previous study that reported that Katana ML presented better BFS and characteristic strength values than Katana STML and UTML after aging, each composed of 3Y-, 4Y-, and 5Y-TZP [27].

Additionally, this study demonstrated the decrease in the nanoindentation hardness and Young’s modulus measured through continuous stiffness testing. Nanoindentation hardness and Young’s modulus tests have been effectively utilized to represent hydrothermally aged zirconia surfaces and have been reported to be effective techniques for evaluating the mechanical properties of small volumes [34,35]. Nanoindentation hardness and Young’s modulus decreased after aging. This is in accordance with a previous study, which reported that the nanoindentation hardness and Young’s modulus of Katana UTML and STML, each composed of 3Y-, 4Y-, and 5Y-TZP, decreased after hydrothermal aging [51]. Moreover, there was a significant decrease in nanoindentation hardness values of ZL and ZP. This was presumed to be due to the induction of microcracks caused by phase transformation after accelerated aging [52]. Microcracks can degrade the hardness and Young’s modulus [53].

The Vickers microhardness values, which are related to abrasion resistance, tended to decrease slightly with age, but no significant differences were exhibited in ZL and ZP. The Vickers hardness of ZP was similar those of ZL, ZM, and ZT before and after aging. These results are similar to those of a previous study by Marchry et al. [54], which indicated that the Vickers hardness values of different zones of multilayered translucent zirconia ceramics were similar.

The tested zirconia ceramics were affected by the effects of the hydrothermal aging process based on the surface analysis data. Surface analyses included surface roughness, phase transformation, surface microstructure, and elemental analysis. Surface roughness must be considered to ensure the long-term success of a material and achieve the desirable esthetic appearance. It was measured using CLSM. CLSM is a ‘reflection’ microscope rather than a conventional fluorescence microscope. As the name suggests, the topographic image is obtained purely through the reflection of the beam incident on the sample, which can be sputter-coated with gold to ensure uniform reflection; usually, the device needs to be reconfigured, and the sample surface is not naturally reflective [55,56,57]. The surface roughness values for all the specimen groups showed an increasing trend with longer aging times. Therefore, the surface of the zirconia ceramics exhibited a proportional increase in roughness as a result of the hydrothermal aging process. 3Y-TZP had greater surface roughness than 4Y- and 5Y-TZP before and after aging. Previous studies have reported similar results, as zirconia stabilized with a normally higher yttrium oxide content has a higher aging resistance, resulting in lower phase transformation, which induces surface roughening [33,58].

Roughness in the range of 0.25 to 0.50 μm has been reported as perceptible by the tongue [31]. The results of the present study showed a significant increase in the roughness of all the zirconia ceramics after aging. However, the *Ra* and *Rq* values were all between 0.003 and 0.007 μm, which are expected to be clinically acceptable as it is lower than the *Ra* value of 0.2 μm.

XRD was used to quantify and evaluate the various phases present on the surface of the zirconia ceramics throughout the aging process. As expected, the aged groups experienced LTD due to the *t⟶m* transformation in ZL and ZP. ZM and ZT were resistant to phase transformations for the applied aging times, and little monoclinic content was presented even after prolonged aging. This result agreed well with earlier findings, as 4Y- and 5Y-TZP revealed significantly reduced phase transformations compared with 3Y-TZP [19,37,59]. According to the XRD results, the monoclinic phase increased with aging time, and after 10 h, the aged groups exhibited monoclinic zirconia at or near the surface. This observation is consistent with previous studies that confirmed phase transformations in other zirconia ceramics after aging [19,38]. Furthermore, t⟶m transformation of the artificially aged zirconia ceramics, as analyzed by XRD, indicated an increase in surface roughness. This is consistent with previous research, as the volume expansion (3–5%) associated with t⟶m transformation leads to grain push-out and surface uplift, resulting in surface roughening [8,9,34,35].

Zirconia ceramics are commonly fabricated using Y-TZP blocks and undergo various surface treatments. In this study, the monoclinic phase was barely evident in the control group. This is attributed to the processing procedures involving a final heat treatment at 900–1000 °C for 1 h for these specimens. The observed results are consistent with several studies that have demonstrated that heat treatments at 900–1000 °C induce a reverse transformation to the tetragonal phase in Y-TZP following sandblasting, grinding, or aging [19,20].

In the FE-SEM and EDS analyses conducted in the present study, slight microcracks, grain push-out, and surface uplift were observed in all the specimens after aging. ZL and ZP exhibited similar results. This was judged to be a result of the particle decrease after aging process and may be related to variations in the surface composition. The surfaces of the aged samples exhibited several ruffled cracks. The surfaces of the aged groups for 10 h exhibited various defects, with slight damage to the internal structure. These observations are consistent with previous studies that confirmed the occurrence of microcracks, surface uplift, and grain push-out as a result of particle decrease in other zirconia ceramics after hydrothermal aging [51,60].

ZP exhibited similar flexural strength, Vickers hardness, phase distribution changes, and surface microstructure changes as ZL before and after aging; however, its surface roughness was lower than that of ZL and higher than those of ZM and ZT after aging.

However, a limitation of this study was the absence of an investigation of the impact of aging on wear and opposing dentition. Moreover, it cannot reproduce various oral environmental factors, such as the influence of chewing forces, salivary components, temperature, and moisture conditions. Further research should be performed to correlate with various in vivo studies to analyze the clinical implications of various properties of conventional and multilayered translucent zirconia ceramics during aging processes. Recently, IPS e.max ZirCAD Prime Esthetic (Ivoclar Vivadent Inc., Schaan, Lichtenstein) has been introduced to the market, which utilizes the gradient technology to form a transition zone by mixing 4Y- and 5Y-TZP as raw materials. Further comparative studies of the 4Y/5Y-TZP transition zone with different zones in multilayered translucent monolithic zirconia ceramics are required.

## 5. Conclusions

Within the limitations of the present in vitro study, the following conclusions were drawn:

1. The flexural strength of 3Y-TZP was higher than those of 4Y-TZP and 5Y-TZP, both before and after aging. However, its stiffness was relatively lower than those of 4Y-TZP and 5Y-TZP.
2. Surface hardness exhibited no significant differences based on yttrium oxide content after aging, indicating similar wear resistances.
3. The surface roughness increased in all the groups after aging, with 3Y-TZP showing the highest, but with clinically acceptable values (*Ra* < 0.2 µm).
4. 3Y/5Y-TZP exhibited flexural strength, Vickers hardness, phase distribution changes, and surface microstructure changes similar to those of 3Y-TZP before and after aging.
5. The mechanical and surface properties of different zones in translucent monolithic zirconia ceramics are expected to be affected by the content of yttrium oxide after hydrothermal aging.

In conclusion, this study presents a novel investigation into the long-term aging effects on the transition zone of multilayered translucent zirconia ceramics, an area that has been largely unexplored in previous research. By analyzing the 3Y/5Y-TZP transition zone fabricated using gradient technology, this study provides unique insights into the long-term structural stability and mechanical performance of multilayered zirconia. The findings indicate that the 3Y/5Y-TZP transition zone exhibits mechanical properties similar to conventional 3Y-TZP, while maintaining enhanced translucency.

These results contribute to the optimization of multilayered zirconia for long-term clinical applications, particularly in anterior restorations where both strength and esthetics are critical. To further enhance the clinical reliability of these advanced materials, future research should explore additional variables, such as fatigue resistance and in vivo degradation. Furthermore, studies on alternative aging conditions and in vivo experiments are necessary to gain a more comprehensive understanding of the long-term performance and clinical relevance of multilayered translucent zirconia ceramics.

## Figures and Tables

**Table 1:** Composition and other details of the tested materials provided by the manufacturers.

Product Name	Manufacturer	Composition (wt%)	Shade (Size)	Lot Number
IPS e.max ZirCAD LT	Ivoclar Vivadent	Zirconium oxide (88.0–95.5 wt%) Yttrium oxide (4.5–6.0 wt%) Hafnium oxide (≤5.0 wt%) Aluminium oxide (≤1.0 wt%) Other oxides (≤1.0 wt%)	2 (Ø98.5 × 18.0 mm)	Z01R0E
IPS e.max ZirCAD MT	Ivoclar Vivadent	Zirconium oxide (86.0–93.5 wt%) Yttrium oxide (6.5–8.0 wt%) Hafnium oxide (≤5.0 wt%) Aluminium oxide (≤1.0 wt%) Other oxides (≤1.0 wt%)	A2 (Ø98.5 × 18.0 mm)	Z01WN7 Z02BMV
IPS e.max ZirCAD MT Multi	Ivoclar Vivadent	Zirconium oxide (86.0–93.5 wt%) Yttrium oxide (6.5–8.0 wt%) Hafnium oxide (≤5.0 wt%) Aluminium oxide (≤1.0 wt%) Other oxides (≤1.0 wt%)	A2 (Ø98.5 × 20.0 mm)	Z02TJS Z031ST
IPS e.max ZirCAD Prime	Ivoclar Vivadent	Zirconium oxide (88.0–95.5 wt%) Yttrium oxide (4.5–7.0 wt%) Hafnium oxide (≤5.0 wt%) Aluminium oxide (≤1.0 wt%) Other oxides (≤1.5 wt%)	A2 (Ø98.5 × 25.0 mm)	Z03FTB

**Table 2:** Abbreviations for the groups of tested materials.

Zone of Material (Yttrium Oxide Content)	Material Code	Groups		
		Control	First Aged	Second Aged
3Y-TZP in IPS e.max ZirCAD LT	ZL	ZLC	ZLAF	ZLAS
4Y-TZP in IPS e.max ZirCAD MT	ZM	ZMC	ZMAF	ZMAS
5Y-TZP in IPS e.max ZirCAD MT Multi	ZT	ZTC	ZTAF	ZTAS
3Y/5Y-TZP in IPS e.max ZirCAD Prime	ZP	ZPC	ZPAF	ZPAS

Y-TZP, Yttria-stabilized tetragonal zirconia polycrystal.

## Data Availability

The original contributions presented in the study are included in the article; further inquiries can be directed to the corresponding author.

## Supplementary Materials

The following supporting information can be downloaded at: https://www.mdpi.com/article/10.3390/jfb16030096/s1, Figure S1: Mean ± standard deviation values and statistical analysis of surface roughness (*S_a_* and *S_q_*) of all specimens in the groups; Table S1: Mean ± standard deviation values and statistical analysis of biaxial flexural strength; Table S2. Weibull moduli and characteristic strengths (MPa) of all the groups; Table S3B. Mean ± standard deviation values and statistical analysis of Young’s modulus; Table S4. Mean ± standard deviation values and statistical analysis of Vickers hardness; Table S5. Mean ± standard deviation values and statistical analysis of surface roughness (Ra and Rq) using CLSM; Table S6. The monoclinic fraction (Fm) was analyzed from the XRD data according to aging time; Table S7A. Mean ± standard deviation and statistical elemental analysis of the IPS e.max ZirCAD LT groups using EDS; Table S7B. Mean ± standard deviation and statistical elemental analysis of the IPS e.max ZirCAD MT groups using EDS; Table S7C. Mean ± standard deviation and statistical elemental analysis of the IPS e.max ZirCAD MT Multi groups using EDS; Table S7D. Mean ± standard deviation and statistical elemental analysis of the IPS e.max ZirCAD Prime groups using EDS; Table S8A. 2-way ANOVA of all groups for mechanical properties values; Table S8B. 2-way ANOVA of all groups for surface roughness of CLSM; Table S8C. 2-way ANOVA of all groups for surface roughness using AFM; Table S8D. 2-way ANOVA of all groups for elemental analysis value of EDS.

## Abbreviations

The following abbreviations are used in this manuscript:

BFS	Biaxial flexural strength
CAD	Computer-aided design
Y-TZP	Yttria-stabilized tetragonal zirconia polycrystal
Y-PSZ	Yttria-partially stabilized zirconia
CLSM	Confocal laser scanning microscopy
XRD	X-ray diffraction
FE-SEM	Field emission scanning electron microscope
EDS	Energy dispersive X-ray spectroscopy
