# Supplementary material for: Changes in the Properties of Different Zones in Multilayered Translucent Zirconia Used in Monolithic Restorations During Aging Process
Source: J Funct Biomater. 2025 Mar 10;16(3):96. doi: 10.3390/jfb16030096 (PMC11943186; doi:10.3390/jfb16030096)
Supplement: Supplementary file 1 [file jfb-16-00096-s001.zip › jfb-3493066-supplementary.pdf]

Journal of
Functional Biomaterials

MDPI

Article

Changes in the Properties of Different Zones in Multilayered Translucent Zirconia Used in Monolithic Restorations During Aging Process

Phil-Joon Koo [1], Jong-Hyuk Lee [1], Seung-Ryong Ha [1], Deog-Gyu Seo [2], Jin-Soo Ahn [3,*] and Yu-Sung Choi [1,4,*]

1 Department of Prosthodontics, College of Dentistry, Dankook University, Cheonan 31116, Republic of Korea; philjoon0310@naver.com (P.-J.K.); hyuk928@dankook.ac.kr (J.-H.L.); hsr@dankook.ac.kr (S.-R.H.)
2 Department of Conservative Dentistry, School of Dentistry and Dental Research Institute, Seoul National University, Seoul 03080, Republic of Korea; dgseo@snu.ac.kr
3 Department of Dental Biomaterials Science and Dental Research Institute, School of Dentistry, Seoul National University, Seoul 03080, Republic of Korea
4 Mechanobiology Dental Medicine Research Center, Dankook University, Cheonan 31116, Republic of Korea
* Correspondence: ahnjin@snu.ac.kr (J.-S.A.); choiys@dankook.ac.kr (Y.-S.C.); Tel.: +82-2-740-8691 (J.-S.A.); +82-41-550-1979 (Y.-S.C.)

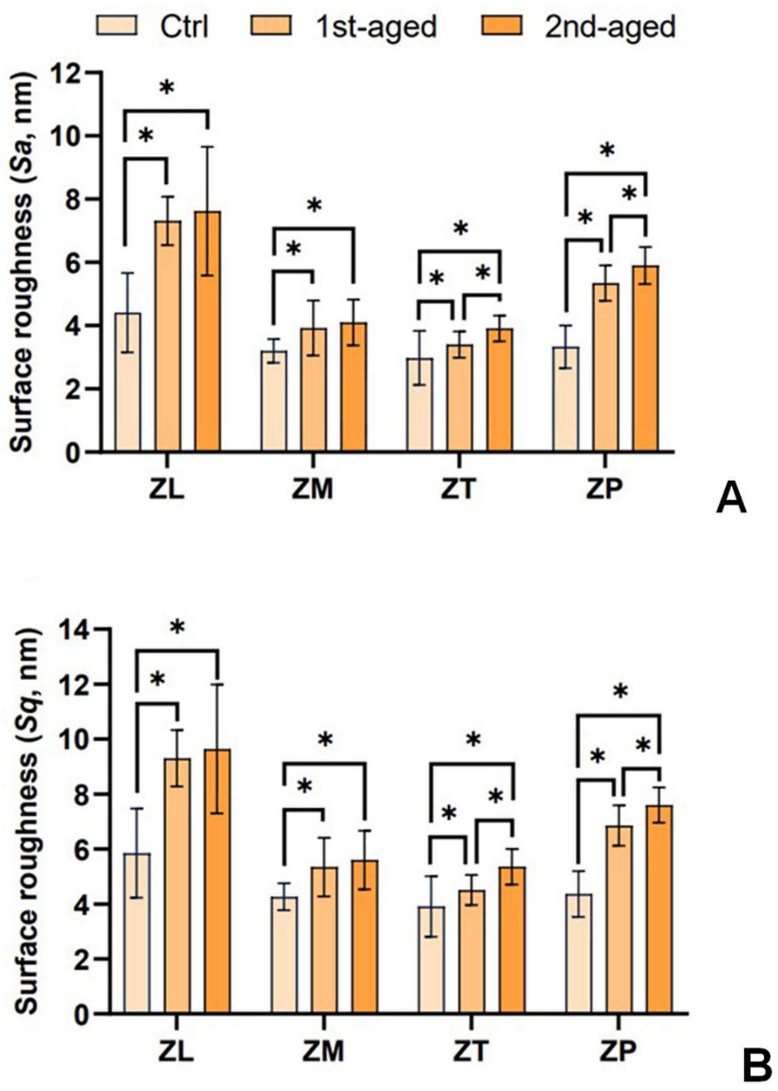

Figure S1. Mean ± standard deviation values and statistical analysis of surface roughness (S_a and S_q) of all specimens in the groups. ZL, 3Y-TZP of IPS e.max ZirCAD LT; ZM, 4Y-TZP of IPS e.max ZirCAD MT; ZT, 5Y-TZP of IPS e.max ZirCAD MT Multi; ZP, 3Y/5Y-TZP of IPS e.max ZirCAD Prime. All data are presented as mean ± SD values. *denotes a significant difference at $P < .05$.

Table S1. Mean ± standard deviation values and statistical analysis of biaxial flexural strength.

Group	Mean ± SD (MPa)					
	Control	First aged	*P*	Second aged	*P* †	*P* ‡
ZL	1102.64 ± 41.37[a]	1096.20 ± 59.17[a]	.733	1059.53 ± 88.29[a]	.102	.192
ZM	841.99 ± 89.67[b]	829.39 ± 103.81[b]	.725	758.69 ± 73.53[b]	.010*	.040*
ZT	827.94 ± 73.86[b]	792.48 ± 99.15[b]	.276	781.09 ± 77.77[b]	.102	.729
ZP	1014.71 ± 139.86[a]	996.91 ± 147.67[a]	.737	956.33 ± 128.64[a]	.344	.429

ZL, 3Y-TZP of IPS e.max ZirCAD LT; ZM, 4Y-TZP of IPS e.max ZirCAD MT; ZT, 5Y-TZP of IPS e.max ZirCAD MT Multi; ZP, 3Y/5Y-TZP of IPS e.max ZirCAD Prime.

Significant differences between the values in each column are denoted by different superscript letters, indicating statistical significance ($P < .05$).

P values were calculated using independent t-test results of samples from the control and first aged groups.

P† values were calculated using independent t-test results of the samples from the control and second aged groups.

P‡ values were calculated using the independent t-test results of the samples of the first and second aged groups.

*denotes a significant difference at $P < .05$.

Table S2. Weibull moduli and characteristic strengths (MPa) of all the groups.

Group	Weibull analysis	Control	First aged	Second aged
ZL	Weibull modulus	31.46	22.14	14.35
	Characteristic strength	1121.63	1122.64	1098.01
ZM	Weibull modulus	10.95	8.37	12.19
	Characteristic strength	881.29	879.26	790.51
ZT	Weibull modulus	12.91	9.46	11.79
	Characteristic strength	861.32	834.50	815.17
ZP	Weibull modulus	8.08	7.06	8.74
	Characteristic strength	1076.88	1066.48	1010.71

ZL, 3Y-TZP of IPS e.max ZirCAD LT; ZM, 4Y-TZP of IPS e.max ZirCAD MT; ZT, 5Y-TZP of IPS e.max ZirCAD MT Multi; ZP, 3Y/5Y-TZP of IPS e.max ZirCAD Prime.

Table S3. A. Mean ± standard deviation values and statistical analysis of nanoindentation hardness.

Group	Mean ± SD (GPa)					
	Control	First aged	*P*	Second aged	*P* †	*P* ‡
ZL	18.30 ± 2.79[a]	11.79 ± 1.80[a]	<.001*	10.61 ± 1.87[a]	<.001*	.021*
ZM	20.82 ± 2.37[b]	19.98 ± 1.67[b]	.135	19.54 ± 2.27[b]	.047*	.426
ZT	20.40 ± 1.80[b]	20.34 ± 2.41[b]	.918	20.29 ± 1.86[b]	.827	.933
ZP	19.40 ± 2.28[ab]	12.48 ± 2.28[a]	<.001*	12.40 ± 1.78[c]	<.001*	.895

ZL, 3Y-TZP of IPS e.max ZirCAD LT; ZM, 4Y-TZP of IPS e.max ZirCAD MT; ZT, 5Y-TZP of IPS e.max ZirCAD MT Multi; ZP, 3Y/5Y-TZP of IPS e.max ZirCAD Prime.

Significant differences between the values in each column are denoted by different superscript letters, indicating statistical significance ($P < .05$).

P values were calculated using independent t-test results of samples from the control and first aged groups.

P† values were calculated using independent t-test results of the samples from the control and second aged groups.

P‡ values were calculated using the independent t-test results of the samples of the first and second aged groups.

*denotes a significant difference at $P < .05$.

Table S3. B. Mean ± standard deviation values and statistical analysis of Young's modulus.

Group	Mean ± SD (GPa)					
	Control	First aged	*P*	Second aged	*P* †	*P* ‡
ZL	263.24 ± 21.81[a]	180.30 ± 17.49[a]	<.001*	158.82 ± 20.09[a]	<.001*	<.001*
ZM	272.37 ± 28.53[ab]	272.29 ± 21.28[b]	.991	257.01 ± 14.83[b]	.018*	.003*
ZT	284.90 ± 20.07[b]	280.80 ± 24.38[b]	.504	271.23 ± 21.85[c]	.020*	.134
ZP	263.29 ± 23.32[a]	191.18 ± 19.37[a]	<.001*	185.99 ± 20.69[d]	<.001*	.345

ZL, 3Y-TZP of IPS e.max ZirCAD LT; ZM, 4Y-TZP of IPS e.max ZirCAD MT; ZT, 5Y-TZP of IPS e.max ZirCAD MT Multi; ZP, 3Y/5Y-TZP of IPS e.max ZirCAD Prime.

Significant differences between the values in each column are denoted by different superscript letters, indicating statistical significance ($P < .05$).

P values were calculated using independent t-test results of samples from the control and first aged groups.

P† values were calculated using independent t-test results of the samples from the control and second aged groups.

P‡ values were calculated using the independent t-test results of the samples of the first and second aged groups.

*denotes a significant difference at $P < .05$.

Table S4. Mean ± standard deviation values and statistical analysis of Vickers hardness.

Group	Mean ± SD (VH)					
	Control	First aged	*P*	Second aged	*P* †	*P* ‡
ZL	1308.86 ± 39.39[a]	1306.57 ± 56.59[a]	.824	1299.42 ± 43.60[a]	.284	.504
ZM	1349.24 ± 64.14[b]	1326.50 ± 71.23[a]	.115	1303.59 ± 36.73[a]	<.001*	.060
ZT	1345.64 ± 66.18[b]	1331.46 ± 68.32[a]	.320	1307.41 ± 69.78[a]	.009*	.102
ZP	1322.86 ± 67.82[ab]	1314.78 ± 71.15[a]	.583	1303.34 ± 58.13[a]	.146	.406

ZL, 3Y-TZP of IPS e.max ZirCAD LT; ZM, 4Y-TZP of IPS e.max ZirCAD MT; ZT, 5Y-TZP of IPS e.max ZirCAD MT Multi; ZP, 3Y/5Y-TZP of IPS e.max ZirCAD Prime.

Significant differences between the values in each column are denoted by different superscript letters, indicating statistical significance ($P < .05$).

P values were calculated using independent t-test results of samples from the control and first aged groups.

P† values were calculated using independent t-test results of the samples from the control and second aged groups.

P‡ values were calculated using the independent t-test results of the samples of the first and second aged groups.

*denotes a significant difference at $P < .05$.

Table S5. Mean ± standard deviation values and statistical analysis of surface roughness (R_a and R_q) using CLSM.

Surface roughness	Group	Mean ± SD (nm)					
		Control	First aged	*P*	Second aged	*P*†	*P* ‡
R_a	ZL	36.45 ± 12.10[a]	92.95 ± 7.41[a]	<.001*	116.75 ± 9.80[a]	<.001*	<.001*
	ZM	38.74 ± 2.59[ab]	40.54 ± 1.39[b]	.003*	41.08 ± 5.84[b]	.049*	.645
	ZT	33.29 ± 3.42[a]	39.34 ± 7.95[b]	<.001*	49.68 ± 7.20[c]	<.001*	<.001*
	ZP	48.69 ± 5.09[c]	61.56 ± 3.87[c]	<.001*	68.84 ± 5.62[d]	<.001*	<.001*
R_q	ZL	47.75 ± 15.62[a]	120.22 ± 12.68[a]	<.001*	147.13 ± 11.52[a]	<.001*	<.001*
	ZM	50.03 ± 4.06[a]	52.96 ± 3.67[b]	.007*	54.19 ± 7.20[b]	.012*	.436
	ZT	45.40 ± 5.19[a]	51.35 ± 10.13[b]	.010*	66.00 ± 10.89[c]	<.001*	<.001*
	ZP	63.94 ± 6.38[b]	79.30 ± 5.19[c]	<.001*	88.98 ± 7.80[d]	<.001*	<.001*

ZL, 3Y-TZP of IPS e.max ZirCAD LT; ZM, 4Y-TZP of IPS e.max ZirCAD MT; ZT, 5Y-TZP of IPS e.max ZirCAD MT Multi; ZP, 3Y/5Y-TZP of IPS e.max ZirCAD Prime.

Significant differences between the values in each column are denoted by different superscript letters, indicating statistical significance (P < .05).

P values were calculated using independent t-test results of samples from the control and first aged groups.

P† values were calculated using independent t-test results of the samples from the control and second aged groups.

P‡ values were calculated using the independent t-test results of the samples of the first and second aged groups.

*denotes a significant difference at P < .05.

Table S6. Mean ± standard deviation values and statistical analysis of surface roughness (R_a, R_q, S_a and S_q) using AFM.

Surface roughness	Group	Mean ± SD (nm)					
		Control	First-aged	*P*	Second-aged	*P*†	*P* ‡
R_a	ZL	3.95 ± 0.34^a	6.70 ± 0.62^a	<.001*	6.92 ± 0.80^a	<.001*	.260
	ZM	3.07 ± 0.43^b	3.66 ± 0.94^b	.005*	3.99 ± 0.71^b	<.001*	.154
	ZT	2.95 ± 0.86^b	3.38 ± 0.42^b	.026*	3.87 ± 0.37^b	<.001*	<.001*
	ZP	3.31 ± 0.66^b	5.33 ± 0.55^c	<.001*	5.87 ± 0.59^c	<.001*	<.001*
R_q	ZL	5.44 ± 0.85^a	8.82 ± 1.08^a	<.001*	8.90 ± 1.04^a	<.001*	.781
	ZM	4.05 ± 0.53^b	5.08 ± 1.12^b	<.001*	5.40 ± 1.06^b	<.001*	.299
	ZT	3.90 ± 1.12^b	4.51 ± 0.55^b	.016*	5.34 ± 0.64^b	<.001*	<.001*
	ZP	4.36 ± 0.83^b	6.84 ± 0.73^c	<.001*	7.58 ± 0.67^c	<.001*	<.001*
S_a	ZL	4.41 ± 1.25^a	7.31 ± 0.76^a	<.001*	7.62 ± 2.03^a	<.001*	.462
	ZM	3.20 ± 0.38^b	3.92 ± 0.87^b	<.001*	4.10 ± 0.72^b	<.001*	.418
	ZT	2.98 ± 0.86^b	3.40 ± 0.41^b	.026*	3.91 ± 0.41^b	<.001*	<.001*
	ZP	3.33 ± 0.68^b	5.34 ± 0.56^c	<.001*	5.90 ± 0.58^c	<.001*	<.001*
S_q	ZL	5.86 ± 1.62^a	9.30 ± 1.03^a	<.001*	9.64 ± 2.34^a	<.001*	.488
	ZM	4.27 ± 0.49^b	5.34 ± 1.07^b	<.001*	5.60 ± 1.07^b	<.001*	.384
	ZT	3.91 ± 1.10^b	4.51 ± 0.55^b	.016*	5.36 ± 0.65^b	<.001*	<.001*
	ZP	4.37 ± 0.83^b	6.86 ± 0.73^c	<.001*	7.60 ± 0.64^c	<.001*	<.001*

ZL, 3Y-TZP of IPS e.max ZirCAD LT; ZM, 4Y-TZP of IPS e.max ZirCAD MT; ZT, 5Y-TZP of IPS e.max ZirCAD MT Multi; ZP, 3Y/5Y-TZP of IPS e.max ZirCAD Prime.

Significant differences between the values in each column are denoted by different superscript letters, indicating statistical significance (P < .05).

P values were calculated using independent t-test results of samples from the control and first aged groups.

P† values were calculated using independent t-test results of the samples from the control and second aged groups.

P‡ values were calculated using the independent t-test results of the samples of the first and second aged groups.

*denotes a significant difference at P < .05.

Table S7. The monoclinic fraction (F_m) was analyzed from the XRD data according to aging time.

Group	Monoclinic fraction (F_m, %)		
	Control	First aged	Second aged
ZL	2.47	3.96	4.02
ZM	2.59	2.61	2.63
ZT	2.46	2.47	2.49
ZP	2.56	3.80	3.98

ZL, 3Y-TZP of IPS e.max ZirCAD LT; ZM, 4Y-TZP of IPS e.max ZirCAD MT; ZT, 5Y-TZP of IPS e.max ZirCAD MT Multi; ZP, 3Y/5Y-TZP of IPS e.max ZirCAD Prime.

Table S8. A. Mean ± standard deviation and statistical elemental analysis of the IPS e.max ZirCAD LT groups using EDS.

Group	Mean ± SD (wt%)				
	Zr	O	Y	Hf	Al
ZLC	65.63 ± 0.31^{a}	29.33 ± 0.49^{a}	3.32 ± 0.22^{a}	1.66 ± 0.07^{a}	0.06 ± 0.02^{a}
ZLAF	65.71 ± 0.34^{a}	29.29 ± 0.46^{a}	3.31 ± 0.20^{a}	1.63 ± 0.13^{a}	0.06 ± 0.04^{a}
P	.338	.764	.862	.269	.429
ZLAS	65.51 ± 0.21^{ab}	29.43 ± 0.19^{a}	3.34 ± 0.18^{a}	1.66 ± 0.09^{a}	0.07 ± 0.05^{a}
P†	.098	.334	.748	.921	.674
P ‡	.010*	.156	.590	.337	.351

ZL, 3Y-TZP of IPS e.max ZirCAD LT; ZM, 4Y-TZP of IPS e.max ZirCAD MT; ZT, 5Y-TZP of IPS e.max ZirCAD MT Multi; ZP, 3Y/5Y-TZP of IPS e.max ZirCAD Prime.

Significant differences between the values in each column are denoted by different superscript letters, indicating statistical significance (P < .05).

P values were calculated using independent t-test results of samples from the control and first aged groups.

P† values were calculated using independent t-test results of the samples from the control and second aged groups.

P‡ values were calculated using the independent t-test results of the samples of the first and second aged groups.

*denotes a significant difference at P < .05.

Table S8. B. Mean ± standard deviation and statistical elemental analysis of the IPS e.max ZirCAD MT groups using EDS.

Group	Mean ± SD (wt%)				
	Zr	O	Y	Hf	Al
ZMC	68.37 ± 0.24^{a}	24.61 ± 0.21^{a}	5.26 ± 0.09^{a}	1.72 ± 0.08^{a}	0.04 ± 0.03^{a}
ZMAF	68.93 ± 0.13^{b}	24.09 ± 0.15^{b}	5.19 ± 0.14^{b}	1.74 ± 0.10^{a}	0.04 ± 0.02^{a}
P	<.001*	<.001*	.034*	.557	.415
ZMAS	68.49 ± 0.23^{a}	24.50 ± 0.18^{c}	5.31 ± 0.20^{a}	1.66 ± 0.08^{b}	0.04 ± 0.02^{a}
P†	.071	.044*	.279	.007*	.573
P ‡	<.001*	<.001*	.018*	.004*	.694

ZL, 3Y-TZP of IPS e.max ZirCAD LT; ZM, 4Y-TZP of IPS e.max ZirCAD MT; ZT, 5Y-TZP of IPS e.max ZirCAD MT Multi; ZP, 3Y/5Y-TZP of IPS e.max ZirCAD Prime.

Significant differences between the values in each column are denoted by different superscript letters, indicating statistical significance (P < .05).

P values were calculated using independent t-test results of samples from the control and first aged groups.

P† values were calculated using independent t-test results of the samples from the control and second aged groups.

P‡ values were calculated using the independent t-test results of the samples of the first and second aged groups.

*denotes a significant difference at P < .05.

Table S8. C. Mean ± standard deviation and statistical elemental analysis of the IPS e.max ZirCAD MT Multi groups using EDS.

Group	Mean ± SD (wt%)				
	Zr	O	Y	Hf	Al
ZTC	66.80 ± 0.19^{a}	24.56 ± 0.19^{a}	6.95 ± 0.15^{a}	1.64 ± 0.10^{a}	0.05 ± 0.02^{a}
ZTAF	66.83 ± 0.10^{a}	24.32 ± 0.10^{b}	7.07 ± 0.20^{b}	1.74 ± 0.10^{b}	0.04 ± 0.02^{a}
P	.588	<.001*	.021*	<.001*	.702
ZTAS	66.74 ± 0.13^{ab}	24.47 ± 0.11^{c}	7.06 ± 0.16^{b}	1.65 ± 0.09^{a}	0.08 ± 0.03^{b}
P†	.176	.047*	.016*	.705	<.001*
P ‡	.012*	<.001*	.843	<.001*	<.001*

ZL, 3Y-TZP of IPS e.max ZirCAD LT; ZM, 4Y-TZP of IPS e.max ZirCAD MT; ZT, 5Y-TZP of IPS e.max ZirCAD MT Multi; ZP, 3Y/5Y-TZP of IPS e.max ZirCAD Prime.

Significant differences between the values in each column are denoted by different superscript letters, indicating statistical significance (P < .05).

P values were calculated using independent t-test results of samples from the control and first aged groups.

P† values were calculated using independent t-test results of the samples from the control and second aged groups.

P‡ values were calculated using the independent t-test results of the samples of the first and second aged groups.

*denotes a significant difference at P < .05.

Table S8. D. Mean ± standard deviation and statistical elemental analysis of the IPS e.max ZirCAD Prime groups using EDS.

Group	Mean ± SD (wt%)				
	Zr	O	Y	Hf	Al
ZPC	69.90 ± 0.16[a]	24.83 ± 0.18[a]	3.45 ± 0.13[a]	1.80 ± 0.08[a]	0.02 ± 0.02[a]
ZPAF	69.81 ± 0.16[a]	24.82 ± 0.15[a]	3.58 ± 0.22[b]	1.76 ± 0.09[a]	0.03 ± 0.02[a]
P	.060	.942	.014*	.090	.838
ZPAS	69.89 ± 0.20[a]	24.90 ± 0.31[a]	3.39 ± 0.25[a]	1.79 ± 0.16[a]	0.04 ± 0.02[b]
P†	.791	.288	.2211	.745	.003*
P ‡	.158	.246	.004*	.396	.002*

ZL, 3Y-TZP of IPS e.max ZirCAD LT; ZM, 4Y-TZP of IPS e.max ZirCAD MT; ZT, 5Y-TZP of IPS e.max ZirCAD MT Multi; ZP, 3Y/5Y-TZP of IPS e.max ZirCAD Prime.

Significant differences between the values in each column are denoted by different superscript letters, indicating statistical significance (P < .05).

P values were calculated using independent t-test results of samples from the control and first aged groups.

P† values were calculated using independent t-test results of the samples from the control and second aged groups.

P‡ values were calculated using the independent t-test results of the samples of the first and second aged groups.

*denotes a significant difference at P < .05.
